# Supplementary material for: Association between the ABCC11 gene polymorphism-determined earwax properties and external auditory canal microbiota in healthy adults
Source: Microbiol Spectr. 2025 Jan 16;13(2):e01698-24. doi: 10.1128/spectrum.01698-24 (PMC11792512; doi:10.1128/spectrum.01698-24)
Supplement: Supplemental material — Fig. S1 to S4. [file spectrum.01698-24-s0001.pdf]

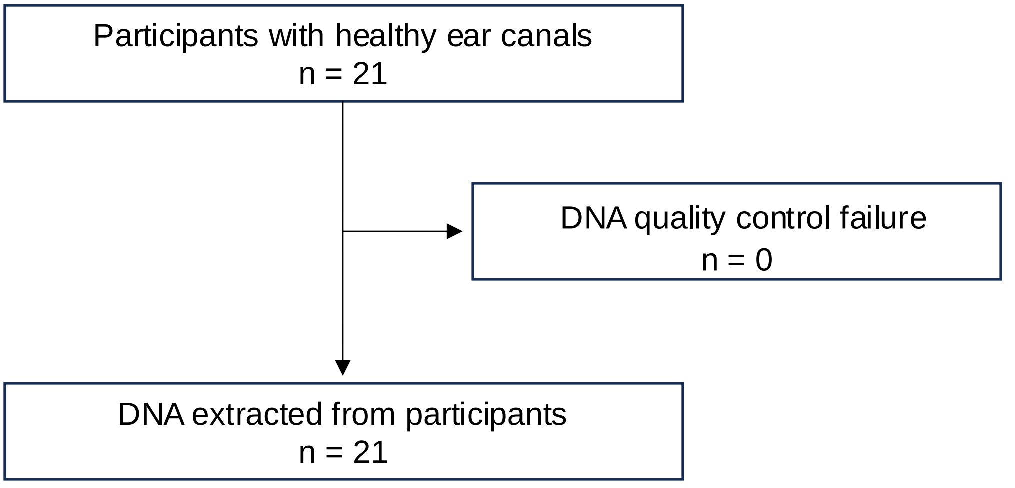

Figure S1. Participant selection.
Patients with concomitant ear disease, malignant disease, or a history of treatment with antimicrobial or steroid preparations within one month were excluded. Samples were collected from the left external ear canal of all 21 participants for bacterial microbiota analysis.

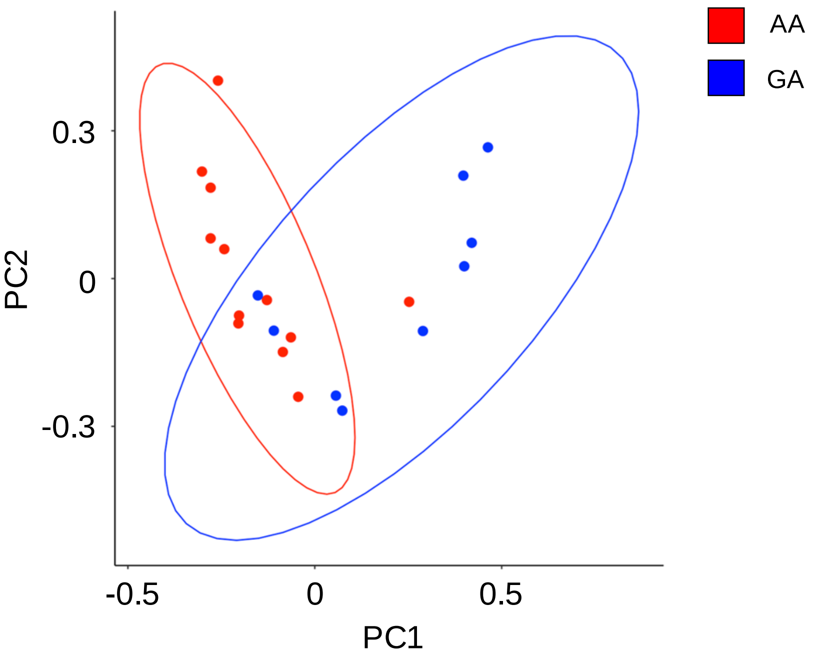

Figure S2. Association of ABCC11 gene polymorphisms with the bacterial microbiota of the external auditory canal.
Beta diversity in the ear canal bacterial microbiota of healthy participants (9 in the AA group and 12 in the GA group) analyzed using QIIME2. The clusters are circled.

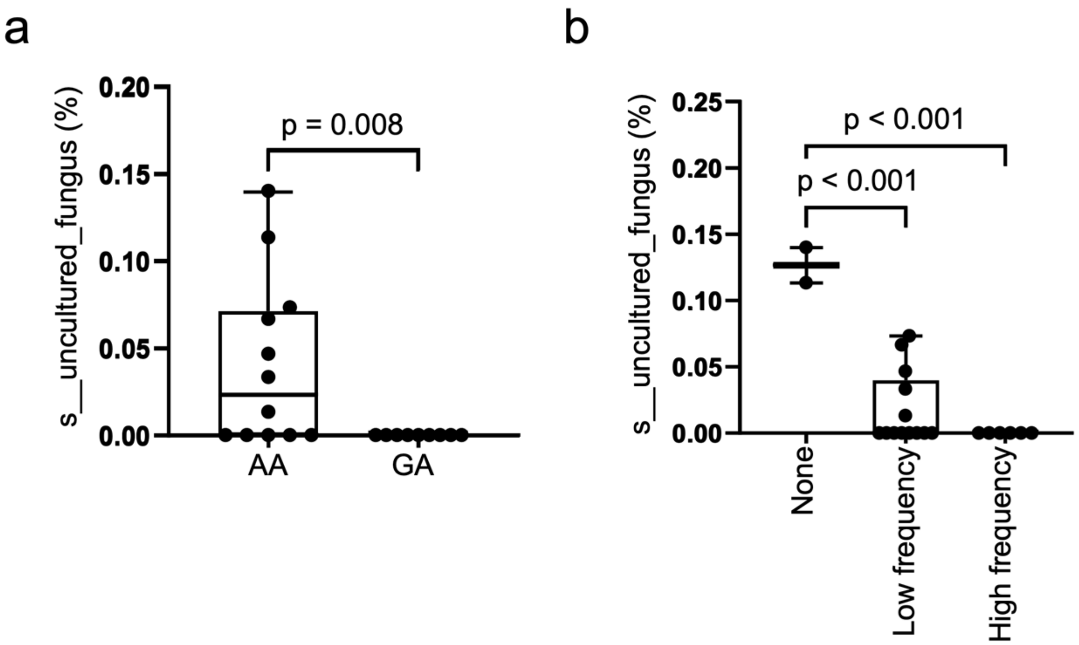

Figure S3. Differences in the uncultured fungus of the ear canal owing to *ABCC11* gene polymorphisms and ear-cleaning habits.

a. Comparison of the relative abundance of uncultured fungus in the AA and GA groups.
b. Comparison of the relative abundance of uncultured fungus by ear-cleaning habits.

One-way analysis of variance [ANOVA] with Tukey–Kramer's HSD test was used to compare ear-cleaning habits and the proportions of each microbiota.

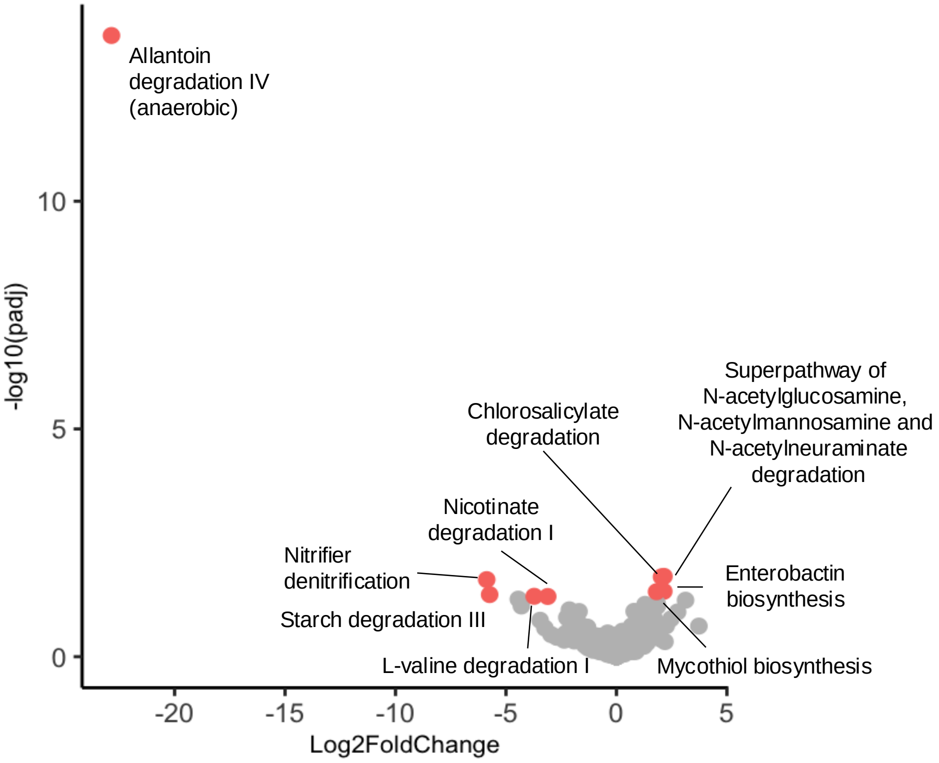

Figure S4. PICRUSt predicts bacterial flora function.
Volcano plot demonstrating functional predictions of the bacterial microbiota. This graph demonstrates various functional predictions between the two groups. Red dots indicate significant differences between the groups.
